# Crossing Barriers: PEGylated Gold Nanoparticles as Promising Delivery Vehicles for siRNA Delivery in Alzheimer’s Disease

**DOI:** 10.3390/biomedicines13092108

**Published:** 2025-08-29

**Authors:** Elżbieta Okła, Marcin Hołota, Sylwia Michlewska, Serafin Zawadzki, Katarzyna Miłowska, Javier Sánchez-Nieves, Rafael Gómez, Francisco Javier De la Mata, Maria Bryszewska, Maksim Ionov

**Affiliations:** 1 Department of General Biophysics, Faculty of Biology and Environmental Protection, University of Lodz, Pomorska 141/143, 90-236 Lodz, Polandkatarzyna.milowska@biol.uni.lodz.pl (K.M.);; 2 Doctoral School of Exact and Natural Sciences, University of Lodz, 21/23 Matejki St., 90-237 Lodz, Poland; 3 Department of Plant Ecophysiology, Faculty of Biology and Environmental Protection, University of Lodz, Banacha 12/16, 90-237 Lodz, Poland; marcin.holota@biol.uni.lodz.pl; 4 Laboratory of Microscopic Imaging and Specialized Biological Techniques, Faculty of Biology and Environmental Protection, University of Lodz, Banacha 12/16, 90-237 Lodz, Poland; 5 BioMedChem Doctoral School, University of Lodz and Lodz Institutes of the Polish Academy of Sciences, 21/23 Matejki St., 90-237 Lodz, Poland; 6 Departamento de Química Orgánica y Química Inorgánica, Facultad de Farmacia, Universidad de Alcalá, 28805 Alcalá de Henares, Spain; javier.sancheznieves@uah.es (J.S.-N.); javier.delamata@uah.es (F.J.D.l.M.); 7 Networking Research Center on Bioengineering, Biomaterials and Nanomedicine (CIBER-BBN), 28029 Madrid, Spain; 8 Faculty of Medicine, Collegium Medicum, Mazovian Academy in Plock, Pl. Dabrowskiego 2, 09-402 Plock, Poland

**Keywords:** PEGylated gold nanoparticles, siRNA delivery, Human Brain Endothelial Cells

## Abstract

**Background**: The proportion of people suffering from neurodegenerative conditions, such as Alzheimer’s disease (AD), is increasing in the population year on year. Despite the constant effort of researchers, these conditions remain incurable and can only be managed by alleviation or delaying of symptoms. The lack of suitable treatment is caused by constricted access to the brain, limited by the brain-blood barrier. The aim of this work was to investigate two pegylated gold nanoparticles as potential carriers of therapeutic siRNA and their impact on the cellular functions of Human Brain Endothelial Cells. **Methods and Results**: Nanoparticles AuNP14a and AuNP14b complexed with siRNA were internalized by HBEC-5i cells and located in the cytoplasm. The genotoxicity assay proved that the nucleus was not affected and complexed nanoparticles did not cause DNA damage. The reactive oxygen species formation and mitochondrial membrane potential changes were measured and showed an adaptive response of cells after compound administration. Results obtained in a cytotoxicity assay conducted on astrocytes and pericytes, which are components of the blood–brain barrier, confirmed the biosafety of tested nanoparticles. **Conclusions**: In summary, it was shown that AuNP14a and AuNP14b are promising candidates as nanocarriers for therapeutic nucleic acids through biological barriers.

## 1. Introduction

Disorders affecting the nervous system include a wide range of conditions that can prominently impair life quality or preclude independent functioning. According to the 2021 global report, 43% of the world population is affected by neurological conditions [1]. The treatment of neurodegenerative diseases is limited by a constricted access to the structures affected by these conditions. The blood–brain barrier (BBB) and blood–cerebrospinal fluid barrier (BCB) fulfill their functions by protecting the brain and spinal cord from toxins and microorganisms and securing a homeostatic environment by simultaneously hindering drug adsorption and metabolism [2]. All these limitations caused that, despite advanced biotechnology, Alzheimer’s disease (AD), a disorder of cognitive functions caused by nerve cell damage, is still incurable. Up to date, only seven drugs that delay or reduce its symptoms are available on the market. These include two monoclonal antibodies against specific forms of β-amyloid and five drugs with the activity of acetylcholinesterase inhibitors or NMDA receptor antagonists [2,3]. Moreover, limitation of drug–drug interactions in AD therapy, caused by patients frequent coexisting conditions such as hypertension, obesity, or type 2 diabetes mellitus, involves a continuous need for innovative therapeutic approaches [4]. Alzheimer’s disease onset is correlated to carrying at least one allele of the ε4 isoform of the apolipoprotein E coding gene [5]. *APOE4* is associated with the intraneuronal accumulation of β-amyloid and its constricted clearance, and it is also connected with weaker Aβ phagocytosis, tau protein aggregate exacerbation, and impairment of lipid and glucose metabolism [6]. Therefore, targeting and modulating APOE has been considered in possible AD therapeutic strategies. This includes the use of the anti-*APOE4* short interfering RNA (siRNA) as a silencer of the defective gene. The major advantage of siRNA therapy is its high specificity, which makes it a promising alternative to small molecule drugs or proteins [7]. In the case of Alzheimer’s disease, a few molecular targets are aimed at as therapeutic options, e.g., Aβ levels may be decreased by silencing the presenilin 1 or *BACE1* gene, and axonal regeneration may be induced by *ROCK2* silencing [8].

However, naked siRNA is not very efficient due to its instability, insufficient cell uptake, and off-target effects [9]. These limitations could be overcome by introducing formulation modifications based on viral and non-viral structures. The non-viral structures include a wide variety of liposomes, polymers, peptides, or inorganic compounds [10]. To date, six siRNA agents have been approved by the FDA. Five of them are conjugated with N-acetylgalactosamine (GalNAc), and one, Patisiran, used in polyneuropathy, is formulated as a lipid nanoparticle (LNP), proving the efficacy of the implemented modifications [11,12].

Among inorganic compounds that could be used as siRNA carriers are nanoparticles of gold (AuNPs). These cationic structures have a multitude of virtues, such as stability, low toxicity and size-to-volume ratio, which enable the binding and transporting of the desired molecules [13]. Their bioavailability, efficacy, and safety can be improved by various surface modifications, namely by cell-penetrating peptides (CPPs), poly(β-amino ester), or polyethylene glycol (PEG) [14].

Our previous research was focused on choosing optimal dendron generation and dendron/PEG ratio for PEG-modified AuNPs [15]. The aim was to obtain non-toxic and efficient nanocarriers, which are able to penetrate cells. Two gold nanoparticles, AuNP14a and AuNP14b with dendron/PEG ratios of 3:1 and 1:1, respectively, were marked as promising candidates. They were initially verified for their biosafety and ability to complex siRNA [16]. Their interactions with lipid membranes and selected serum proteins were also described [17]. In this work we focused on the properties of 14a and 14b as carriers for siRNA, testing uptake and the effects on internalized cells.

## 2. Materials and Methods

### 2.1. AuNPs and siRNA

Two pegylated gold nanoparticles, AuNP14a and AuNP14b, were synthesized and described in previous work [15]. Both AuNPs were decorated with a second generation of ammonium-terminated carbosilane dendrons and PEG fragments in different ratios. AuNP14a contained a 3:1 ratio dendron/PEG while AuNP14b had a ratio of 1:1. AuNPs were synthesized in water by the reaction of HAuCl4·3H2O with a mixture of two ligands containing a thiol moiety: (1) the cationic dendrons HSG_2_(SNMe^3+^)_4_ and (2) commercial PEG ligand CH_3_O(CH_2_CH_2_O)_n_CH_2_CH_2_SH, HS-PEG. NaBH_4_ was used as a reducing agent. Their characteristics was described in Table 1.

Gold nanoparticles were complexed with non-fluorescent or FITC-labeled siRNA coding ApoE (sense: 5′-GAUUACCUGCGCUGGGUGCUU-3′; antisense: 5′-PGCACCCAGCGCAGGUAAUCUU-3′) purchased from Dharmacon Inc. (Lafayette, CO, USA). AuNPs/siRNA complexes were formed by mixing appropriate volumes of siRNA and AuNPs in PBS. The mixture was gently vortexed and incubated at room temperature for 20 min. Complex formation was proved by a series of biophysical experiments, including zeta potential and DLS measurements, agarose gel electrophoresis, and fluorescence polarization. Main characteristics are shown in Table 2.

Nanoparticle concentration selected for the complex formation was assessed in preliminary studies by biophysical experiments. A wide concentration range was checked to choose the optimal amount of AuNPs to form complexes and to see at which point they may be harmful.

### 2.2. Cell Lines

Human Brain Endothelial Cell (HBEC-5i) line was purchased from the American Type Culture Collection (ATCC, Manassas, VA, USA). The cells were cultured on 1% gelatin-coated flasks in Dulbecco’s Modified Eagle Medium/Nutrient Mixture F12 (DMEM-F12) (Biowest, Nuaillé, France) and supplemented with 10% heat-inactivated fetal bovine serum (FBS), 1% penicillin/streptomycin (P/S), Endothelial Cell Growth Supplement (ECGS) (Sigma-Aldrich, Darmstadt, Germany), and 1 µg/mL hydrocortisone (MP Biomedicals, Santa Ana, CA, USA) at 37 °C, 5% CO_2_.

Human Brain Vascular Pericytes (HBVP) line was purchased from ScienCell Research Laboratories (Carlsbad, CA, USA). The cells were cultured on flasks coated with 2 µg/cm^2^ Poly-L-Lisyne (PLL) in a pericyte medium supplemented with FBS, pericyte growth supplement (PGS), and P/S (ScienCell Research Laboratories, Carlsbad, CA, USA) at 37 °C, 5% CO_2_.

Human astrocytes (HA) line was purchased from ScienCell Research Laboratories (Carlsbad, CA, USA). The cells were cultured on flasks coated with 2 µg/cm^2^ Poly-L-Lysine (PLL) in an astrocyte medium supplemented with FBS, astrocyte growth supplement (AGS), and P/S (ScienCell Research Laboratories, Carlsbad, CA, USA) at 37 °C, 5% CO_2_.

### 2.3. Cytotoxicity

The cytotoxic effects of AuNPs and AuNP/siRNA complexes toward astrocytes and pericytes were assessed with an MTT assay. Cells were seeded on 96-well plates at a density of 1 × 10^4^ cells/well and incubated under standard conditions. The following day, cells were treated with the tested compounds. After 24 h, 0.5 mg/mL MTT solution (3-(4, 5-dimethylthiazol-2-yl)-2,5-diphenyltetrazolium bromide) was added to the cells and incubated for 3 h. Formazan crystals formed as a product of tetrazolium dye reduction were dissolved in DMSO (Avantor Performance Materials Poland S.A., Gliwice, Poland) and measured for absorbance at the wavelengths λ = 580 nm and λ = 720 nm on a multiwell plate reader (BioTek PowerWave HT, BioTek Instruments, Inc., Winooski, VT, USA). The cell viability was calculated using the following formula:

% viability = (As/Ac) × 100%

where As—absorbance of treated cells, Ac—absorbance of control cells.

### 2.4. Genotoxicity

The DNA damage induced by the AuNPs/siRNA complexes in HBEC-5i cells was assessed using the comet assay. The HBEC-5i cell line was seeded on a 24-well plate at a density of 5 × 10^4^ cells/well and collected after 24 h of incubation with nanoparticles and their complexes with siRNA. Next, the cells were collected, washed with PBS, and embedded in a 1% solution of low melting point (LMP) agarose (Sigma-Aldrich, Darmstadt, Germany). The suspension was set on the top of a microscope slide pre-coated with a normal melting point (NMP) agarose (Sigma-Aldrich, Darmstadt, Germany) and left on ice to congeal. Prepared slides were immersed overnight at 4 °C in a lysis buffer (2.5 mol/L NaCl, 100 mm/L EDTA, 10 mmol/L Tris, and Triton X-100 1%). After 24 h, the slides were placed in a cold electrophoresis buffer (30 mmol/L NaOH, 1 mmol/L EDTA) for 20 min to allow DNA unwinding, then electrophoresis was conducted (17 V, 32 mA, 30 min). Slides were then washed 3× with distilled water and stained with 0.1% DAPI solution. Fluorescence imaging was conducted using a Zeiss Axio Scope A1 fluorescence microscope (Carl Zeiss, Germany). A total of 50–75 randomly selected cell nuclei per slide were analyzed using the Lucia-Comet v. 7.6 software (Laboratory Imaging, Praha, Czech Republic).

### 2.5. Cellular Uptake

The internalization of AuNPs/siRNA complexes by HBEC-5i cells was evaluated using confocal microscopy and flow cytometry. The quantification of nanocomplexes was assessed by measuring fluorescence intensity via flow cytometry. Additionally, confocal microscopy was employed to verify the intracellular presence of the nanocomplexes.

For the confocal microscopy, cells were incubated with free siRNA and AuNP/siRNA complexes for 3 h or 24 h. HBEC-5i were seeded in a density of 1.5 × 10^4^ cells/well on the glass bottom plates (Ibidi GmbH, Gräfelfing, Germany) and cultured under standard conditions. The next day, cells were treated with complexes. The cells were then fixed with 4% formaldehyde (Avantor Performance Materials Poland S.A., Gliwice, Poland). The samples were washed with PBS (GibcoTM, ThermoFischer Scientific, Waltham, MA, USA) and stained with Texas Red-X Phalloidin (40 diluted) (Invitrogen ™, ThermoFischer Scientific, Waltham, MA, USA) for 20 min and DAPI (4′,6-diamidino-2-phenylindole) (Thermo Scientific ™, ThermoFischer Scientific, Waltham, MA, USA) at 0.5 μg/mL for 5 min. Samples were visualized using a Leica TCS SP8 confocal microscope (Leica Microsystems, Wetzlar, Germany) equipped with a 63×/1.40 objective (HC PL APO CS2, Leica Microsystems, Germany). The excitation and emission wavelengths were set as follows: 405 nm and 430–470 nm for DAPI, 489 nm and 500–530 nm for FITC ™ 488, and 595 nm with 610–640 nm for Texas Red-X Phalloidin. Image analysis was performed using Leica LAS 2.0.215022 software.

Quantitative data was obtained by the flow cytometry technique. HBEC-5i cells were seeded on a 24-well plate at a density of 5 × 10^4^ cells/well. The next day, cells were treated with complexes and incubated for 3 h or 24 h. The cells were then washed 2× with PBS and collected in round-bottom tubes. The fluorescence intensity of FITC-labeled siRNA was measured using a Becton Dickinson LSRII flow cytometer (Becton Dickinson, Franklin Lakes, NJ, USA) with excitation/emission wavelength set at 488/530 nm. Data was analyzed using FCSalyzer 0.9.22-alpha software.

### 2.6. Reactive Oxygen Species Formation

The level of reactive oxygen species (ROS) formed in HBEC-5i cells after AuNPs and AuNPs/siRNA treatment was measured using the fluorescence method. In this assay a 2′, 7′-dichlorodihydrofluorescein diacetate (H2DCF-DA) fluorescent probe was used (Thermo Fisher Scientific, Waltham, MA, USA). H2DCF-DA was internalized to cells by passive diffusion and degraded by membrane esterases to more polar DCFH. When ROS was present in the environment, DCFH was oxidated to fluorescent DCF.

HBEC-5i cells were seeded on black 96-well plates at a density of 1.5 × 10^4^ cells/well and incubated under standard conditions. After 24 h, cells were treated with nanoparticles or complexes. ROS formation was assessed at 3 different incubation times, after 0.5 h, 3 h, and 24 h. Cells were then washed with PBS and incubated with an H2DCF-DA probe at the final concentration of 2 µmol/L for 15 min at 37 °C. Using the multiwell plate reader (BioTek PowerWave HT, BioTek Instruments, Inc., Winooski, VT, USA), fluorescence was measured at λex = 485 nm and λem = 530 nm.

### 2.7. Mitochondrial Membrane Potential

The measurements of the mitochondrial membrane potential (∆Ψm) were made using fluorescent probe JC-1 (Thermo Fisher Scientific, Waltham, MA, USA). The dye can emit both green and red fluorescence depending on its form. When mitochondrial functions were impaired, the JC-1 stayed in the monomeric form, giving green fluorescence (filters 485/540 nm). Red fluorescence (filters 530/590 nm) was emitted by the JC-1 aggregates created by healthy cells. Thus, a high ratio of red to green fluorescence can be correlated to high polarization of the mitochondrial membrane.

HBEC-5i cells were seeded on black 96-well plates at a density of 1.5 × 10^4^ cells/well and incubated under standard conditions. After 24 h, cells were treated with nanoparticles or complexes. The experiment was conducted at 3 different time points: after 0.5 h, 3 h, and 24 h. Cells were washed with PBS and incubated with the JC-1 solution at a final concentration of 5 µmol/L for 20 min at 37 °C. Fluorescence was measured on the multiwell plate reader (BioTek PowerWave HT, BioTek Instruments, Inc., Winooski, VT, USA) at λex = 485 nm, λem = 530 nm for monomers and λex = 528 nm, λem = 590 nm for dimers.

### 2.8. Statistical Analysis

Statistical data and their graphical representations were prepared using GraphPad Prism 8.0.2 Software. The Shapiro–Wilk normality test was used to assess the Gaussian distribution for each experiment. Statistical significance was assumed as α = 0.05, calculated with one-way ANOVA (cellular uptake), Kruskal–Wallis (comet assay), or Dunnett’s multiple comparison tests (ROS and mitochondrial activity assays, cytotoxicity tests).

## 3. Results

### 3.1. Complex Cellular Uptake

**Figure 1:**
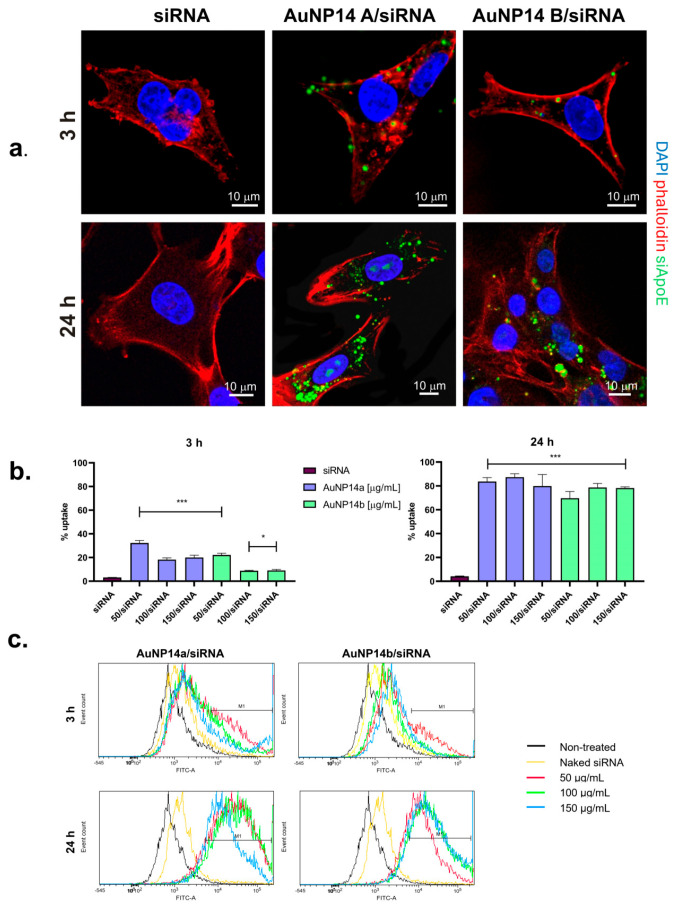
Cellular uptake of AuNPs/siRNA complexes. (**a**) Confocal microscopy images of HBEC-5i cells treated with tested complexes at 3 h and 24 h incubation times. Images represent one chosen concentration of nanoparticles (50 µg/mL). Complexes internalization was more visible after 24 in case of both nanoparticles. (**b**) Graphs showing mean fluorescence values (MFI) of AuNPs/siRNA measured with flow cytometry. Results are compared with cells treated with naked siRNA. Obtained data is consistent with microscopy images. Values represent mean ± SD, * *p* < 0.05, *** *p* < 0.01. (**c**) Flow cytometry histograms rendered using FCSalyzer software.

Complexes of both nanoparticles with siRNA were tested for their internalization abilities using quantitative flow cytometry. The uptake of AuNPs/siRNA was compared after 3 h and 24 h incubation with HBEC-5i cells. It was observed that in both time periods, siRNA was delivered to cells; however, longer incubation was conducive to more abundant internalization (Figure 1b,c). After 3 h, the uptake of complexes was already noticeable and fluctuated around 20%, and after 24 h, this value reached around 80%. These results corresponded to the qualitative confocal microscopy images, where fluorescently labeled siRNA was visible as a single form in short-time incubation slides and as thickset aggregates in 24 h slides (Figure 1a).

### 3.2. Genotoxicity

**Figure 2:**
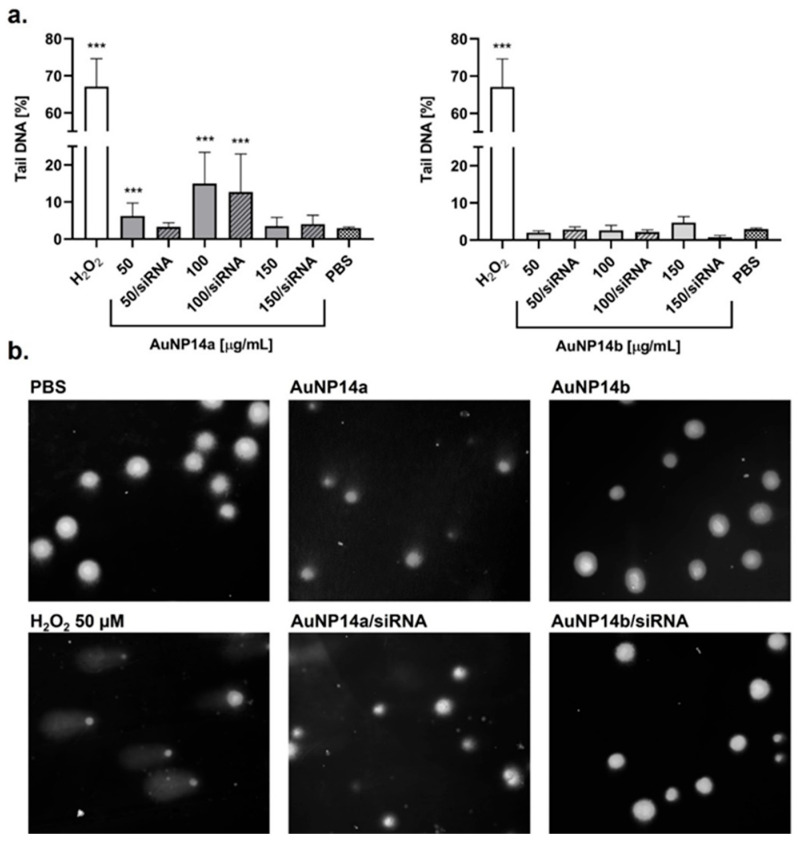
Genotoxic effect caused by AuNPs and AuNPs/siRNA in HBEC-5i cells. (**a**) Percentage of DNA damage in treated cells after 24 h incubation with tested compounds. Cells treated with 50 µM H_2_O_2_ for 15 min at 37 °C were a positive control. Results indicate that genotoxic effect was observed only when higher concentrations of AuNP14a were applied. Values shown as median ± CI 95% from at least 50 nucleloids per slide, *** *p* < 0.01. (**b**) Images taken with fluorescent microscope (20× magnification) showing nucleus of cells stained with DAPI after treatment with AuNPs and AuNPs/siRNA in chosen concentrations.

The alkaline comet assay was used to assess the cell nucleus damage after AuNPs and AuNPs/siRNA treatment. DNA disruption was shown as a percentage of the comet tail and corresponded to the leakage of genetic material outside the nucleus. Data was collected after 24 h incubation with tested compounds, and results were compared with the cells treated with 50 µM H_2_O_2_ that represented the positive control. Both tested nanoparticles proved to be less genotoxic when they were complexed with siRNA, showing a DNA disruption at levels below 10%, comparable with the negative control (Figure 2). AuNP14a, having more of a cationic load than AuNP14b (higher positively charged dendron to neutral PEG ratio), was more likely to cause DNA damage, particularly in higher concentrations. However, at 50 µg/mL, where complexation and internalization both occur, both nanoparticles were safe for siRNA.

### 3.3. Reactive Oxygen Species and Mitochondrial Membranes Potential Changes

Reactive oxygen species (ROS) are formed during normal metabolic processes, but their production is also linked with cell responses to different substances introduced to the organism. When this response is the excessive overproduction of ROS, it can lead to oxidative stress and cell disruption. High levels of ROS can alter the polarization of mitochondrial membranes (∆Ψm), causing metabolic dysfunction, or apoptosis. Therefore, monitoring these two parameters could provide information about the cell response to a delivered compound.

**Figure 3:**
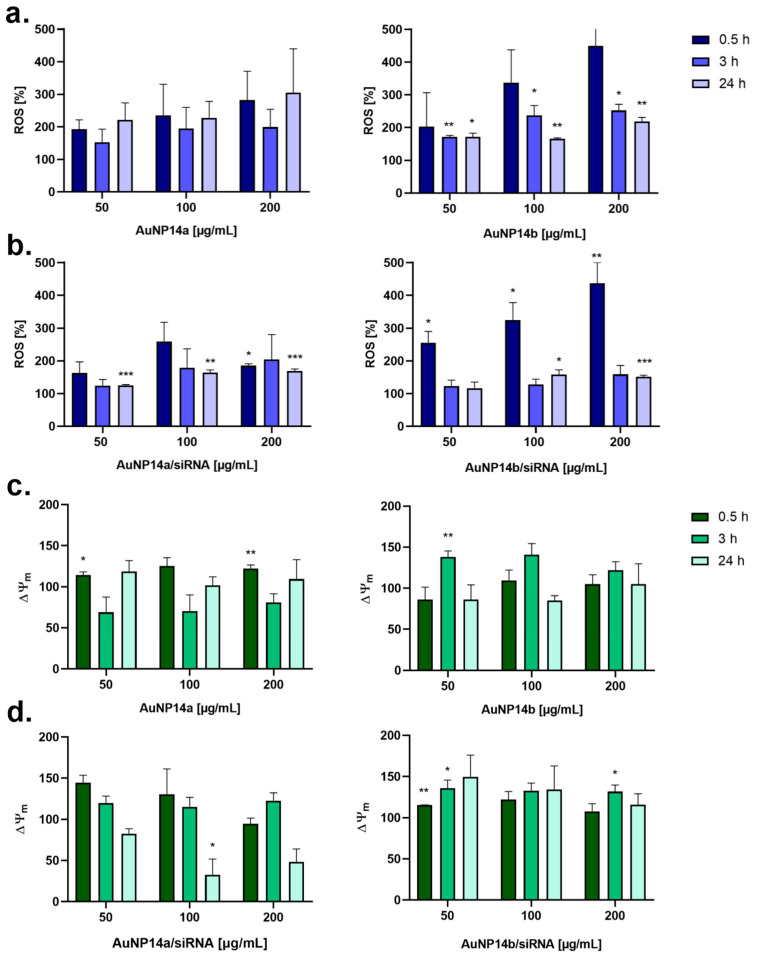
Changes in reactive oxygen species level (**a**,**b**) and mitochondrial membrane polarization (**c**,**d**) in HBEC-5i cells after AuNPs and AuNPs/siRNA administration. Graphs show mean ± SD, n = 3, * *p* < 0.05, ** *p* < 0.03, *** *p* < 0.01. Untreated cells (control) are considered as 100% ROS production.

For better understanding of these processes, ROS production and ∆Ψm at different time intervals (0.5 h, 3 h, and 24 h) were measured. In general, it was observed that the ROS level increased rapidly after the administration of tested compounds and stabilized in time (Figure 3). This dependency was notably visible in the case of the treatment with complexes, where any ROS formation caused by a lower dosage was re-established to basic levels after 3 h and was stable after 24 h (Figure 3b). A similar pattern applied to changes in the mitochondrial membrane potential. Alterations in membrane polarization were most evident after a 3 h incubation but only increased or decreased an insignificant amount after 24 h nanoparticle or complex treatment (Figure 3c,d).

### 3.4. AuNPs Cytotoxicity Towards Human Brain Vascular Pericytes and Astrocytes

**Figure 4:**
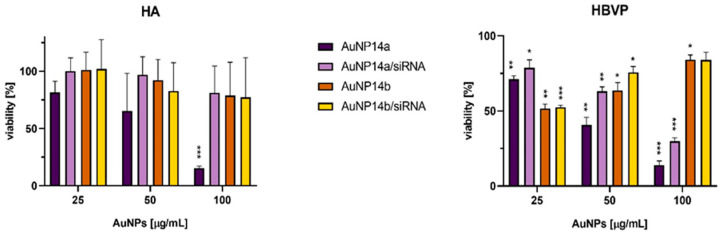
Viability of HA and HBVP after 24 h incubation with AuNPs and their complexes with siRNA. Non-treated cells were taken as a 100% viability control. Graphs show mean ± SD, n = 3, * *p* < 0.05, ** *p* < 0.03, *** *p* < 0.01.

The cytotoxic effects of AuNPs and their complexes were previously described for HBEC-5i cells [13]. However, it was decided to extend the cytotoxicity analysis for further research concerning BBB interactions. The viability of human astrocytes (HA) and human brain vascular pericytes (HBVP) after 24 h incubation with AuNPs and AuNPs/siRNA using the MTT assay was tested (Figure 4). AuNP14a alone caused the most significant cytotoxic effect in both cell lines in a concentration-dependent manner, whereas complexation with siRNA moderated the effect, especially in lower concentrations. AuNP14b seemed to be more harmful in lower doses, particularly for HBVP cells. Contrastingly, the highest concentrations of both AuNP14b and its complex were safe for tested cell lines.

## 4. Discussion

The effective delivery of drugs in the management of neurodegenerative diseases, such as Alzheimer’s disease, still remains a big challenge for researchers and clinicians [18]. The scientists are examining different approaches for finding the safest and most beneficial solution by analyzing invasive (e.g., intracerebral injections) and non-invasive (e.g., carrier-drug conjugation) routes of administration [19]. This study focused on pegylated gold nanoparticles, which are non-invasive colloidal nanocarriers that could be used as delivery agents of short-interfering RNA targeted at silencing the apolipoprotein Eε4 gene related to Alzheimer’s disease onset.

In our previous research, complex formation and cytotoxic effects for epithelial cells were defined. It was proved that lower concentrations of tested AuNPs are safe and may be further analyzed [16]. In this article the ability of these structures to internalize cells was shown. Quantitative data obtained with flow cytometry and supported by the visualization with confocal laser scanning microscopy showed an uptake of AuNPs/siRNA by the HBEC-5i cell line. While internalization was noticeable after 3 h incubation, the biggest effect was seen after 24 h, when cells were full of fluorescently labeled siRNA and flow cytometry analysis indicated around an 80% uptake. Observation of the fluorescence peaks on flow cytometry diagrams (Figure 1c) supported the chosen complex concentrations, showing the highest FITC intensity for the lower dose of AuNP14a (50 µg/mL) and the higher dose of AuNP14b (100 µg/mL). Cationic nanoparticles, due to their biophysical properties, are, in general, excellent delivery systems and materials that complex negatively charged nucleic acids. Taschauer et al. compared the uptake of both positively and negatively charged auropolypexes, proving a significantly lower internalization of the latter [20]. Interestingly, nucleic acid delivery systems with a gold nanoparticle core underwent a human phase 0 study with positive effects, where sufficient uptake levels and confirmed biosafety were fundamental [21]. Analyses of microscopy images suggest that complexes did not enter the nucleus but accumulated in the cytoplasm, where the actual gene silencing takes place [22]. Avoiding nucleus internalization is a desirable property of nanoparticles for their effectiveness but also for minimizing their genotoxic potential [23].

Genotoxicity testing of nanomaterials is highly recommended during uptake studies. It is important to correlate the obtained data with cytotoxicity results to identify any true exogenous DNA damage, apart from any low level of DNA disruption occurring in the early stages of apoptosis [24]. In order to confirm that the tested compounds have no adverse effects on genetic material and the cell nucleus, the alkaline comet assay was performed. This method not only allows us to visualize the level of DNA damage but also to present the data in figures by using dedicated software. After 24 h incubation of cells with AuNPs, we observed that AuNP14a created some genotoxic effect when not complexed with siRNA (Figure 2). At the highest concentrations, the level of DNA damage decreased, but that phenomenon was aligned with the cytotoxicity assay and, as mentioned above, could have been a sign of early apoptosis [16]. Fraga et al. presumed that the failure to induce genotoxicity by higher AuNPs concentration may also be the result of possible nanoparticle aggregation [25]. However, complexation with siRNA mitigated the genotoxic effect of AuNP14a, and the complex was proven to be safe for genetic material. AuNP14b caused no significant genotoxic effect when compared to the control, regardless of whether it was administrated as a complex or not. The genotoxicity of gold nanoparticles has been previously described as being strongly dependent on their biophysical properties, mainly size. The size of tested AuNP14a and AuNP14b is 34 nm and 23.3 nm, respectively. That may be an important advantage compared to other gold nanoparticles, due to the fact that some researchers have indicated that smaller NPs are prone to being more genotoxic [26,27,28]. Bigger AuNP14a particles appeared to cause more damage in the DNA strands than smaller AuNP14b; therefore, we confronted obtained results with reactive oxygen species formation. Any triggering of genotoxicity by nanoparticles could be described by primary and secondary mechanisms. While the primary mechanism is related to the direct interaction with DNA and ROS formation, the secondary is associated with evoking innate immunity cells and inflammatory reactions leading to free radical production [29].

ROS accumulation in HBEC-5i cells was measured using the H2DCF-DA probe at three different time points: after 0.5 h, 3 h, and 24 h. Since ROS production can cause mitochondrial damage, the obtained results were interpreted in juxtaposition to mitochondrial membrane polarization changes (ΔΨm) [30]. In general, both nanoparticles were less prone to induce reactive species production when complexed with siRNA, although according to the literature, AuNPs with PEG or PEI generated oxidative stress at lower levels than those without functionalization [31]. AuNP14a caused composite ROS formation, with the results equivocal and difficult to interpret (Figure 3). However, polarization oxidative stress conditions were verified after comparing the results to changes in mitochondrial membrane polarization, where AuNP14a prompted a hyperpolarization of mitochondrial membranes after 0.5 h of incubation and visible membrane depolarization within the next 3 h. The most rapid increase in ROS level was observed for AuNP14b/siRNA, where after 0.5 h the percentage of oxidative forms rose 3–4×, afterward stabilizing to a neutral level. Simultaneously, the mitochondrial membrane potential was the nearest to its natural state shortly after the complex administration and increased in time, suggesting an adaptive response at first, including SOD enzyme activation dealing with stress conditions, and subsequently enhanced mitochondrial activity [32]. The AuNP14a/siRNA generated ROS production in a similar manner as AuNP14b, but the mitochondrial membrane polarization alterations were inverted and decreased over time, which could indicate mitochondrial damage. AuNPs can cause oxidative stress not only by triggering mitochondrial impairing or DNA disruption, but it has also been been seen that gold nanoparticles are able to catalyze electron donors such as antioxidants, consequently leading to ROS formation [33]. This mechanism could be useful while testing AuNPs as antibacterial agents [34]. Taking everything into consideration, gold nanoparticles described in the literature could cause oxidative stress with insignificant changes in cell physiology or could be used as radiosensitization in tumors, where redox potential is crucial and the obtained effect is mainly dependent on the AuNPs coating and their size [35,36].

In our previous work, the cytotoxic effect of tested compounds toward the epithelial cell line HBEC-5i has been described. Here, we tested whether AuNPs and their complexes were safe for two other cell lines: human astrocytes (HA) and human brain vascular pericytes (HBVP), as these are used in the standard triculture BBB in vitro model [37,38]. It was observed that both nanoparticles were less cytotoxic when complexed with siRNA, which remains consistent with the HBEC-5i results and genotoxicity analysis. This correlation could have been the effect of surface charge equalization between cationic AuNPs and negatively charged siRNA [39]. Smaller AuNP14b particles decreased the viability of both cell lines more noticeably than larger AuNP14a particles when applied in smaller concentrations. However, this result agreed with the conclusions of other researchers and could be explained by the smaller particle aggregation in cytoplasm, leading to endosomal or lysosomal entrapment and pH alteration induced toxicity [40,41].

These results provided the foundation for further analysis of AuNPs as siRNA carriers. Firstly, they present new information about cells response to tested compounds. Moreover, oxidative stress, mitochondrial function loss, or impaired cellular uptake play a crucial role in the pathology of neurodegenerative diseases; thus, it is crucial to check whether tested nanocarriers will enhance or reduce these effects [42]. Gold nanoparticles are widely investigated as siRNA delivery vehicles by many researchers. It was proved that their multiple modification options, size variety, or synthesis techniques may provide new features of perfect nanocarriers. AuNP14a and AuNP14b described in this work meet the requirements of high quality gold nanoparticles, such as biocompatibility and effective cellular uptake, which makes them promising candidates for gene material delivery systems. However, it is crucial to emphasize that in vitro models, while valuable, cannot fully replicate the complexity of in vivo conditions. Therefore, any future prospect of research must include BBB triculture model experiments and in vivo tests, as these methods may help us to explain the lack of current knowledge.

## 5. Conclusions

Our studies showed that AuNP14a/siRNA and AuNP14b/siRNA were able to internalize HBEC-5i cells in a concentration- and time-dependent manner. Additionally, although an increase in the ROS level after AuNPs administration was observed in both complexes, there was no DNA damage seen. By correlating free radical production to changes in the mitochondrial membrane polarization, it was concluded that any stress condition was an adaptive reaction to the nanoparticles and stabilized within 24 h. Despite the fact that AuNPs/siRNA were found to be safe for endothelial cells, astrocytes, and pericytes (the cell lines forming the BBB), there is a necessity for more developed models for testing these complexes, such as triculture experiments and in vivo methods. Future perspectives of our studies include testing BBB model permeability and immunological response for AuNPs/siRNA, along with biodistribution in vivo tests, supported by behavior assessment. It is crucial considering many clinical challenges, such as PEG-induced rapid clearance or hypersensitivity reactions caused by anti-PEG antibody activation [43]. The questions of gold hypothetical accumulation in the tissues or manufacturing reproducibility should also be addressed to provide high quality of the tested compounds [44]. Comprehensive data obtained from any next stage will show if AuNP14a and AuNP14b are the efficient and harmless perfect nanocarriers.

## Figures and Tables

**Table 1:** Characterization of tested AuNPs. ^1^ Zeta potential (mV); ^2^ diameter (nm) obtained by DLS; ^3^ Polydispersity index (PDI) obtained by DLS; ^4^ diameter obtained by TEM; ^5^ % organic matter obtained by TGA, corresponding with dendron and PEG.

AuNP	Solubility	Dendron/PEG Molar Ratio	^1^ ZP [mV]	^2^ d_z_ [nm]	^3^ PDI	^4^ d_n_ [nm]	^5^ %L
AuNP14a	water	3/1	+44.9	34.00	0.54	3.7	67.6
AuNP14b	water	1/1	+41.1	23.3	0.298	2.8	66.6

**Table 2:** Characterization of tested AuNPs/siRNA. ^1^ Concentration of siRNA; ^2^ minimal concentration of AuNPs where complexed formation was observed; ^3^ zeta potential (mV); ^4^ diameter (nm) obtained by DLS; ^5^ polydispersity index (PDI) obtained by DLS.

Complex	^1^ siRNA [µM]	^2^ AuNP [µg/mL]	^3^ ZP [mV]	^4^ d_z_ [nm]	^5^ PDI
AuNP14a/siRNA	1	40	+21.97	227.23	0.57
AuNP14b/siRNA	1	50	+7.97	161.53	0.219

## Data Availability

The datasets generated and analyzed during the current study are available from the corresponding author on reasonable request.

## Abbreviations

The following abbreviations are used in this manuscript:

AuNPs	Gold nanoparticles
BACE1	Beta-secretase 1
BBB	Blood–brain barrier
BCB	Blood–cerebrospinal fluid barrier
DAPI	4′,6-diamidino-2-phenylindole
DMSO	Dimethyl sulfoxide
FDA	Food and Drug Administration
GalNAc	N-acetylgalactosamine
HA	Human astrocytes
HBVP	Human brain vascular pericytes
JC-1	5,5′,6,6′-tetrachloro-1,1′,3,3′-tetraethylbenzimidazolylcarbocyanine iodide
NMDA	N-methyl-D-aspartate
PEG	Polyethylene glycol
ROCK2	Rho associated coiled-coil containing protein kinase 2
ROS	Reactive oxygen species
